# Implementing Ultrasound Imaging for the Assessment of Muscle and Tendon Properties in Elite Sports: Practical Aspects, Methodological Considerations and Future Directions

**DOI:** 10.1007/s40279-021-01436-7

**Published:** 2021-03-08

**Authors:** Fabio Sarto, Jörg Spörri, Daniel P. Fitze, Jonathan I. Quinlan, Marco V. Narici, Martino V. Franchi

**Affiliations:** 1 Department of Biomedical Sciences, University of Padova, Padova, Italy; 2 Sports Medical Research Group, Department of Orthopaedics, Balgrist University Hospital, University of Zurich, Zurich, Switzerland; 3 Department of Orthopaedics, University Centre for Prevention and Sports Medicine, Balgrist University Hospital, University of Zurich, Zurich, Switzerland; 4 School of Sport, Exercise and Rehabilitation Sciences, University of Birmingham, Birmingham, UK; 5 National Institute for Health Research, Birmingham Biomedical Research Centre at University Hospitals Birmingham NHS Foundation Trust, Birmingham, UK; 6 CIR-MYO Myology Centre, University of Padova, Padova, Italy

## Abstract

Ultrasound (US) imaging has been widely used in both research and clinical settings to evaluate the morphological and mechanical properties of muscle and tendon. In elite sports scenarios, a regular assessment of such properties has great potential, namely for testing the response to training, detecting athletes at higher risks of injury, screening athletes for structural abnormalities related to current or future musculoskeletal complaints, and monitoring their return to sport after a musculoskeletal injury. However, several practical and methodological aspects of US techniques should be considered when applying this technology in the elite sports context. Therefore, this narrative review aims to (1) present the principal US measures and field of applications in the context of elite sports; (2) to discuss, from a methodological perspective, the strengths and shortcomings of US imaging for the assessment of muscle and tendon properties; and (3) to provide future directions for research and application.

## Key Points

**Table:** 

Assessment of muscle and tendon properties using ultrasound imaging may present many different advantageous applications in an elite sports scenario.
In an elite sports context the application of ultrasound is challenging, and thus several practical and methodological recommendations should be considered for its implementation.

## Introduction

Ultrasound (US) imaging represents a relatively affordable, non-invasive, in-vivo method for examining the morphological and mechanical properties of muscle and tendon [1, 2]. Pioneering studies employing US to study musculoskeletal structure and function were conducted in the 1960s [3]; however, its systematic use became more prominent in the 1990s [4–6]. In particular, brightness mode (B-mode) US technique has become widely adopted to quantify skeletal muscle mass and architecture [4, 6, 7] (and their relation with muscle function [8, 9]) in different populations. More recently, researchers have attempted to quantify muscle and tendon behaviours during dynamic tasks such as walking, running, jumping, cycling and swimming [2, 10, 11]. In addition, advancements in US technology such as the extended-field-of-view technique (EFOV), 3D US, shear-wave elastography (SWE) imaging and developments in data processing have enabled deeper insight into the structural and functional properties of muscles and tendons. All of these new technical developments are associated with various benefits, but also with relevant drawbacks and limitations (for an in-depth review of such aspects we refer the reader to Franchi et al. [1]).

In elite sport settings, the assessment of muscle and tendon anatomical and mechanical properties has a great potential for: (1) testing athletes' status with respect to performance-related factors and their response to training; (2) detecting athletes at higher risks of injury; (3) screening athletes for structural abnormalities related to current (or future) musculoskeletal complaints; and (4) monitoring their return to sport after a musculoskeletal injury. However, in elite sports, the time for such assessments is generally scant, resources and equipment may be limited, and the operators are often not trained for such specific US evaluations. Therefore, the application of US for muscle and tendon assessment in these contexts remains challenging.

Accordingly, this narrative review aims to (1) summarize common principal US measures and field of applications in the context of elite sports; (2) highlight from a methodological perspective the strengths and limitations of US imaging for the assessment of muscle and tendon properties; and (3) provide future directions of research and application. Target audience is sport scientists, researchers, as well as elite sports and sports medicine practitioners.

## Practical Aspects

### Principal Ultrasound-Based Measures

In this section, we present most of the principal indices/measures of muscle–tendon morphological and mechanical properties that can be acquired when using US. Their physiological role is then briefly described from a functional perspective (Table 1).

#### Muscle Architecture

**Fig. 1:**
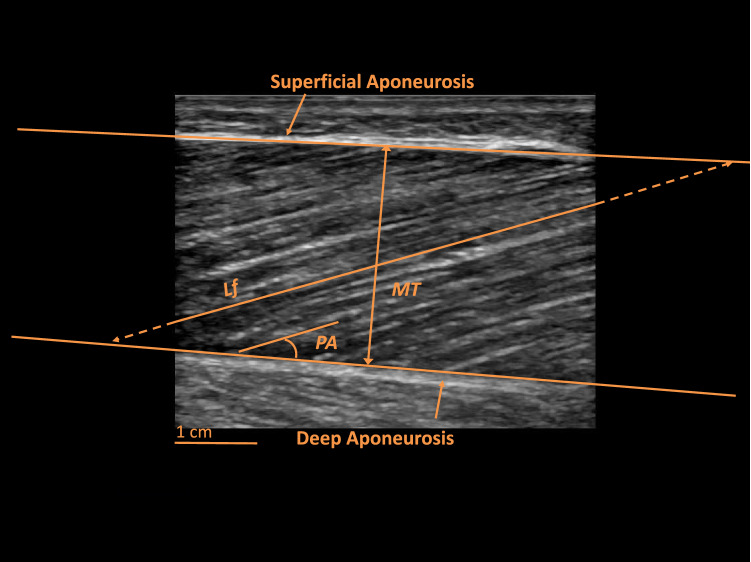
Longitudinal B-mode ultrasound image of a human vastus lateralis muscle captured at 50% of the total femur length. Highlighted are the deep and superficial tendon aponeuroses and the most common features of muscle architecture: *MT* muscle thickness, *Lf* fascicle length, *PA* pennation angle. Image was obtained using a 4.7 cm linear transducer

**Fig. 2:**
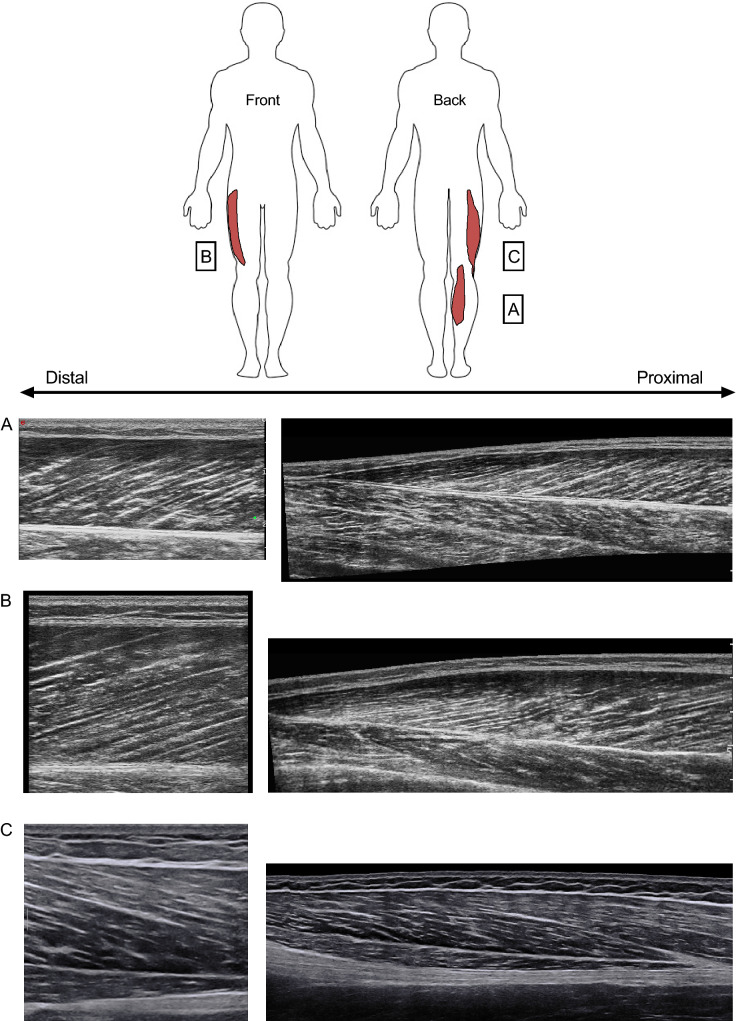
Longitudinal B-mode ultrasound snapshots and respective EFOV scans for three different muscles in young healthy volunteers: *A* vastus lateralis, *B* biceps femoris long head, *C* gastrocnemius medialis. The arrows indicate the distal and proximal muscle portions. Images were obtained using a 4.7 cm linear transducer. *EFOV* extended-field-of-view ultrasound technique

Muscle architecture is defined as the macroscopic arrangement of muscle fibres within a muscle relative to the axis of force generation [8]. It has been previously described as the “structural property of the entire muscle that dominates its function” [8]. When the transducer is correctly aligned with the muscle fascicle plane, we can fully distinguish the orientation of fascicles relative to the transducer due to the presence of high contrast between connective and muscle tissue [10]. Thus, this correct alignment is essential for image quality as it takes advantage of the anisotropic nature of muscle. The length of the muscle fascicles (Lf) and the pennation angle (PA) (i.e., the angle formed by the insertion of the fascicle within the deep tendinous aponeurosis) (Fig. 1), represent the architectural parameters that can be detected by B-mode or EFOV US modalities (Fig. 2) [1, 12]. Lf and PA, determined via B-mode US, are generally reliable and valid as shown in a systematic review by Kwah et al. [13].

Muscle architecture has previously been recognised as one of the primary determinants of muscle function [8]. Theoretically, Lf is dependent on the length and number of sarcomeres in series [8, 14, 15], while PA is mostly seen as a strategy that allows more contractile material to be packed in along the deep aponeurosis of a muscle, seemingly reflecting the number of sarcomeres arranged in parallel [6, 16]. Architectural characteristics can influence muscle force–length and force–velocity relationships [8, 9, 12]. Gans [16] and Lieber [8] explained theoretically that shorter muscle fibres present a lower maximum shortening velocity which likely would influence the velocity of contraction at the entire muscle level [12]. In contrast, the maximum force developed by a muscle is proportional to the number of sarcomeres in parallel, and thus to their PA [12]. However, despite these theoretical considerations, the reports showing a clear linear relationship between functional parameters and muscle architecture in-vivo are controversial, as we discuss in Sect. 3.1.

#### Muscle Dimensions

**Fig. 3:**
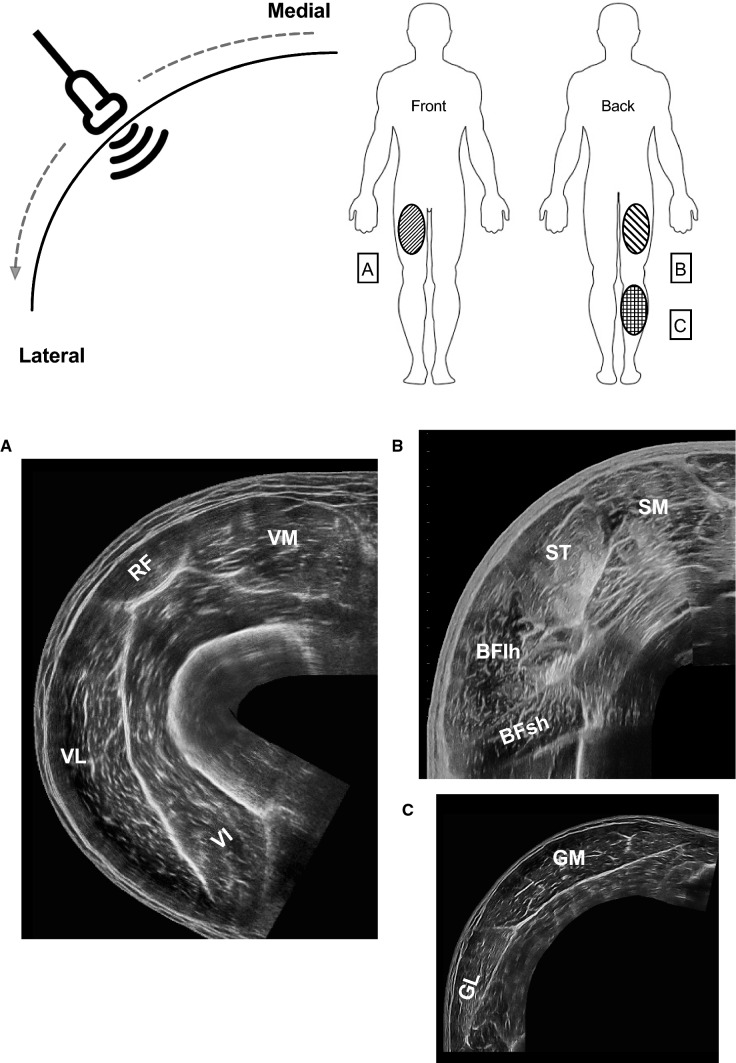
Transverse EFOV scans for three distinct muscle groups in young healthy volunteers: *A* quadriceps femoris, *B* hamstrings, *C* triceps surae. Images were obtained using a 4.7 cm linear transducer. *EFOV* extended-field-of-view ultrasound technique, *VM* vastus medialis, *RF* rectus femoris, *VL* vastus lateralis, *VI* vastus intermedius, *SM* semimembranosus, *ST* semitendinosus, *BFlh* Biceps Femoris long head, *BFsh* Biceps Femoris short head, *GM* gastrocnemius medialis, *GL* gastrocnemius lateralis

Muscle dimensions can be evaluated through linear, two-dimensional, and volumetric indices. Muscle thickness (MT) represents the simplest and most employed measure of muscle dimensions, typically evaluated through B-mode US as the linear perpendicular distance between skeletal muscle interfaces (Fig. 1) [17]. The transducers can be placed transversally [18] or longitudinally [6, 17] in relation to the region of interest (ROI) chosen for a specific muscle or limb. MT has been shown to be reliable for different muscle groups [17, 19]. US has also been employed to assess larger anatomical structures, such as muscle anatomical cross-sectional area (ACSA), whereby scans are acquired in the transversal plane (Fig. 3) [1]. ACSA scans can be obtained by the images-stitching technique [20] or by EFOV US imaging (i.e., panoramic US technique) [21]. EFOV has been shown to be a valid and reliable method for the assessment of quadriceps [22–24], gastrocnemius [24] and hamstrings [25, 26] ACSA when compared with magnetic resonance imaging (MRI) or computed tomography. In addition, the acquisition of an adequate number of ACSAs (generally 5–12 slices) at different portions of muscle length enables the estimation of muscle volume via different approaches [27]. Use of muscle volume has the advantage of accounting for regional differences in muscle dimension, of particular importance for the detection of resistance training-induced changes in muscle mass [17].

The evaluation of muscle dimensions may play a pivotal role in athletic performance or clinical settings. Indeed, muscle ACSA is strongly related to joint torque production and isokinetic strength in different muscle groups [28–30]. Similarly, muscle volume is considered one of the major determinants of joint torque in humans for both upper and lower limbs [31, 32].

#### Muscle Quality

In addition to measures of muscle morphology, estimation of muscle quality (based on tissue composition) can also be obtained by quantitative musculoskeletal US [33]. Echo intensity (or “echogenicity”) is the measure of the reflectivity of sound waves reflected by the tissue. Echogenicity is considered a proxy of muscle composition and in turn it may be used as an index of muscle quality [33, 34]. Connective tissue and fat are more reflective than muscle tissue and thus appear in the image as lighter grey. The largest region of interest excluding aponeuroses is generally detected and then echo intensity measures are calculated as median grayscale values (a 0–255 scale of arbitrary units, with a higher value indicating more hyperechoic material) using programs such as ImageJ or Photoshop [35]. Low echo intensity values are considered to be associated with greater muscle quality; in contrast, high echo intensity values are thought to be related to muscle impairment [33]. US has been shown to be a reliable tool for the assessment of muscle quality as echo intensity in different populations [33], including young athletes [36]. Echo intensity has been mainly used to study muscle quality changes in aging [34], although attempts to apply it in a sports setting have been conducted [37–39].

#### Tendon Dimensions

Tendon dimensions are most commonly represented by tendon thickness and ACSA. Measurements of tendon thickness are typically obtained from longitudinal scans via B-mode in the resting state [40], whereas tendon ACSA can be measured from axial-plane US images taken at different portions of tendon length [41]. A recent review suggested that US measures of tendon dimensions are reliable, both in terms of relative and absolute reliability [42]. Tendon dimensions are related to the distribution of stress within the tendon itself, such that a greater tendon ACSA allows stress to be spread over a larger area and in turn enables greater force to be transmitted through the tissue [43].

#### Tendon Mechanical Properties

**Fig. 4:**
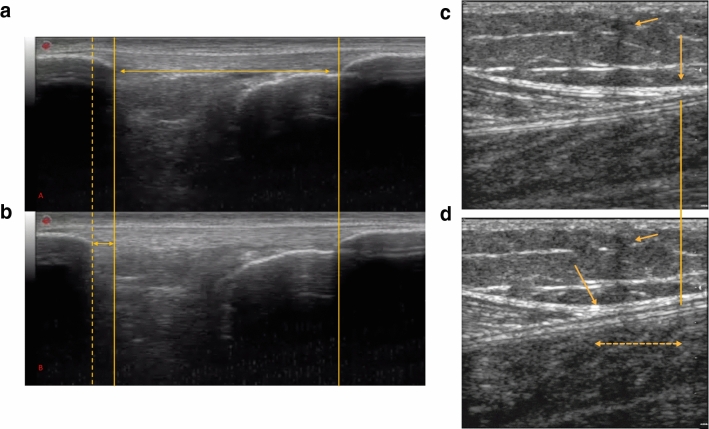
Representative B-mode scans of patellar (**a**, **b**) and Achilles tendon (**c**, **d**). Images are obtained at rest (**a**–**c**) and during isometric ramp contractions (**b**–**d**). The solid lines demonstrate the resting tendon length for the patellar tendon (**a**, **b**) and where the gastrocnemius medialis myotendinous junction is/was situated (**c**, **d**). Dashed lines show the respective elongation of each tendon. The arrows **c**, **d** show the external marker (in this case a wire) that was applied on the skin as a reference. The bidirectional arrow with the broken line **d** indicates the Achilles tendon displacement. Images were obtained using a 4.7 cm linear transducer

The biomechanical properties of tendinous tissues are related to the capacity and effectiveness of these tissues to transmit muscle force to the bone and thus enabling movement [44]. The determination of tendon mechanical properties in vivo has been estimated via the acquisition of force–elongation relationships, such that tendon stiffness is calculated as force divided by tendon elongation [5]. The combination of conventional B-mode US with isometric dynamometry enables the measurement of both tendon deformation (elongation) and force produced during a ramped isometric contraction (Fig. 4). In addition to tendon stiffness (or “elasticity”), other tendon mechanical properties such as strain, stress, Young’s modulus, and hysteresis may be obtained [45]. Unsurprisingly, this non-invasive approach has been widely applied to study tendon plasticity in response to increased [41] or reduced [46] loading conditions, especially in consideration of the contribution of tendon biomechanical properties to rapid torque development [47].

#### Muscle and Tendon Stiffness Measured with Elastography

**Fig. 5:**
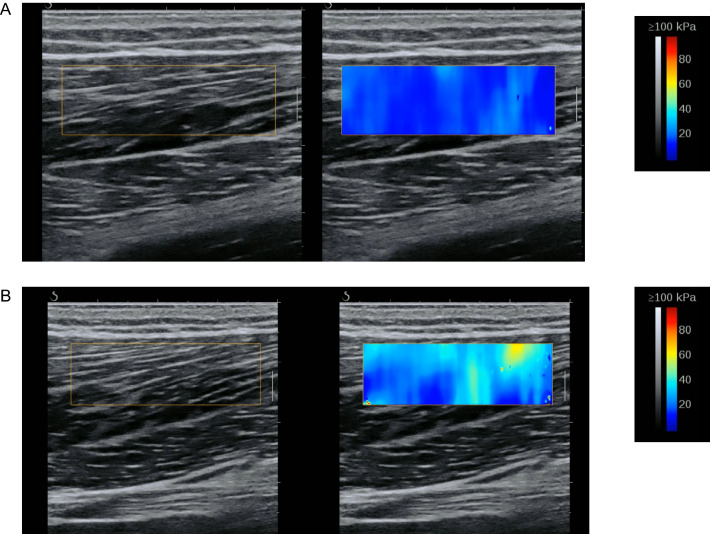
Representative shear wave elastography scan acquired from the biceps femoris long head muscle of two different young healthy volunteers (**a**, **b**). The shear wave modulus elastogram is presented next to the two scans, indicating an increased stiffness for subject B. Images were obtained using a 5 cm linear transducer

The recent introduction of SWE US for the assessment of muscle and tendon mechanical properties allows for the alternative quantifiable estimation of stiffness in the form of an elastogram (Fig. 5) [48, 49]. The most commonly used SWE technique is known as “super-sonic shear imaging” (SSI) [50]. Briefly, the basic principle of SWE is to create an acoustic radiation force impulse displaced in the underlying tissue, resulting in the propagation of a transient shear wave. The instantaneous shear wave velocity obtained (i.e., the calculation of the velocity by which the waves return to the transducer) is directly related to the elastic properties (i.e., shear modulus) of the tissue and can be mapped on an elastogram, which can be also be superimposed onto the B-mode US scan [49, 50]. Similar to many US techniques, SWE is an operator-dependent method; however, when used by an expert operator, it is a reliable tool to evaluate the mechanical properties of different muscles at rest and during passive stretching [51]. Similarly, good reliability was found in the assessment of tendon properties [52].

Quantification of tissue shear modulus has biomechanical, physiological and clinical applications. For instance, muscle stiffness has been shown to be highly related to both active and passive forces and may be used in the near future to reliably estimate the force/torque produced by an individual muscle [50]. Muscle and tendon stiffness are likely to influence also joint mobility (i.e., range of motion) [53]. Moreover, SWE has been used to assess stiffness changes related to training, stretching, manual therapy, dry needling procedures, and injury [48, 49].

## Fields of Application in Elite Sports

### Testing Athletes' Status with Respect to Performance-Related Factors and Their Response to Training

Associations between parameters of muscle architecture/dimensions and sports performance have been frequently observed within various athletic cohorts. Pioneering studies found significant negative correlations between vastus lateralis (VL) and gastrocnemius lateralis Lf and 100-m personal best records in professional male and female sprinters [54, 55]. Similarly, a positive relationship between VL and gastrocnemius lateralis Lf and sprint front crawl swimming performance was observed in young swimmers [56]. Interestingly, VL muscle architecture asymmetry (i.e., the percentage difference between limbs) has been reported to negatively influence jumping power and sprinting speed [37]. Indeed, a recent systematic review and meta-analysis showed an association between jump height with MT but not with lower-limbs muscle architecture [60]. Despite this, there are some studies reporting positive associations between jump height and muscle architecture in athletes belonging to different sports [57–59]. In contrast, a recent study observed a significant relationship between Achilles tendon stiffness and jumping height in children practising artistic gymnastic [61].

Similarly, measures of muscle dimensions have been associated with parameters related to sports performance. Adductors and hamstrings volume (assessed by MRI) [62] and knee extensors MT (medial side, evaluated by US) [63] correlated with sprint performance in highly trained sprinters. Furthermore, biceps femoris long head (BFlh) ACSA obtained by US was associated with 30 m sprint time in young elite football players [64]. VL volume was also found to be a strong predictor of ergometer performance (in a 2000-m time trial), sprint capacity and endurance capacity in Olympic rowers [65]. In long jumpers, ACSA of rectus abdominis (evaluated by MRI) was significantly correlated with long jump performance (i.e., personal long jump performance record) [66]. Significantly, a recent study observed that a larger soleus muscle (increased ACSA and volume) with a thicker Achilles tendon was associated with superior marathon performance [67]. Lastly, quadriceps ACSA showed a robust correlation with weightlifting performance (i.e., maximum one repetition maximum in snatch and clean and jerk) in well-trained female weightlifters [68].

All the observations presented above have been made from cross-sectional studies, and it is important to stress that the reported associations do not provide evidence of causation. Nevertheless, such associations may help in generating potential causal structures that can be tested. Furthermore, in some longitudinal studies, the percentage changes in performance measures has been correlated with the percentage change in morphological musculoskeletal parameters. For example, in a study conducted in female softball players, strong relationships were found between changes in VL muscle architecture (MT and Lf) and changes in sport-specific sprint performance (i.e., time to reach first and second base) over a competitive season [69]. Similarly, young competitive throwers exhibited a significant correlation between the percentage increase in VL MT and percentage increase in a sport-specific throwing test (i.e., the shot put test from the power position), following a tailored-training program [59]. As aforementioned, while the above was of associational nature, these findings seem to support the plausibility of a causal relation.

Overall, associations between sports performance and parameters obtained via US have been consistently reported in such ecologically valid, albeit ‘noisy’ settings. These results should be considered with care, since studies conducted in more controlled laboratory settings demonstrate conflicting results. Indeed, in consideration of the relationships between functional parameters and muscle properties, some report robust associations [70] and others did not observe any [71].

Aside from their association with sports performance, muscle/tendon morphological and mechanical properties are known to be influenced by training. Thus, monitoring these properties over time can be beneficial in an elite sports scenario; namely for the assessment of an individual athlete response to a specific type of training, to the practice of their sport during a season, or throughout their sporting career. Indeed, measurements of training effects are important when monitoring the efficacy of the training stimulus provided to the athletes, and in turn, when controlling and adapting the training program [72]. For instance, it is well known that skeletal muscle hypertrophy occurs in response to strength [14, 73, 74] and plyometric training [75]. Furthermore, different adaptations in muscle architecture [76] have been reported after eccentric [14, 77], concentric [14, 77], isometric [78], sprint [79], plyometric [75, 80] or combined (i.e., plyometric in addition to strength/power training) [81] training modalities. Structural (i.e., ACSA) and mechanical (i.e., stiffness) tendon properties are generally increased after loading interventions [82, 83]. In addition, the isolated practice of a specific sport can induce changes in muscle and tendon morphological and mechanical properties. Differences in muscle dimensions and architecture [84–88] as well as tendon features [85, 86, 89, 90] have been observed in cross-sectional studies comparing athletes practising different sports with non-athletes. Moreover, muscle architecture [91, 92], muscle stiffness [93], tendon stiffness and ACSA [94–96], and capacity to use tendon elastic energy [97] have been shown to differ in relation to chronic sports-specific loading exposure. For the reasons listed above, US assessment has been implemented in longitudinal studies with the aim of investigating changes induced by sports-specific practice during competitive seasons in athletes [69, 90, 98] and/or to investigate the impact of tapering periods [99, 100]. These evaluations may also be of particular interest in youth elite athletes to investigate the combined effects of sports practice and musculoskeletal growth on the functional and morphological development of muscle and tendon structures [101, 102].

### Detecting Athletes at Higher Risks of Injury

It has been proposed that injury occurrence is regulated by a complex mechanical interplay between tissues stress, strain and loading [103]. It follows that alterations in muscle and tendon properties would play a fundamental role in injury processes. Thus, it is unsurprising that different US-derived measures of these parameters have been associated with injury risk in athletes of different sports. In an elite sport scenario, a periodic screening via US may help in estimating a potential risk factors for injury, if the methodological limitations of these studies are taken into account (i.e., lack of evidence of causality).

For instance, muscle Lf has been associated with muscle strain injuries. The theoretical rationale behind this association is that short muscle fascicles may present fewer in-series sarcomeres, and, therefore, may be more susceptible to the over-stretching experienced during powerful eccentric actions such as sprinting [104]. In particular, short biceps femoris long head (BFlh) fascicles have been proposed as a risk factor for hamstrings strain injuries in elite soccer players [104]. Similarly, a retrospective study showed that athletes with a previous hamstrings strain injury have shorter BFlh Lf in the limb with a history of injury than the contralateral uninjured limb [105]. Significantly, a major limitation of these investigations derives from the pitfalls of linear extrapolation methods in the assessment of BFlh Lf, due to the limited field of view (FOV) employed that could have affected the results [106, 107] (the reader is referred to sect. 5.1.2). Therefore, future work should confirm this relationship using more appropriate US methods (e.g., EFOV) or other imaging techniques that would enable a thorough investigation of Lf, such as 3D US or diffusion tensor imaging (DTI).

Measurements of muscle dimensions appear to be related to musculoskeletal injuries. For instance, studies carried out in Australian Football League players have used measures of trunk muscle size to predict musculoskeletal injuries [108–111]. In particular, the ACSA of multifidus muscle [108–111] and the ratio between the ACSAs of the multifidus and quadratus lumborum muscles [111] were related to the occurrence of lower extremities injury both in the pre-season and playing season. In a recent study conducted in young competitive alpine skiers, a smaller lumbar multifidus ACSA (assessed by MRI) was related to a higher occurrence of disc protrusions and end plate alterations [112]. Moreover, bilateral muscle differences in rectus femoris and VL ACSAs may be associated with games missed because of lower extremity injury in professional basketball players [38]. Based on the positive influence on maximal strength capacity [28–32], muscle dimensions measures assessed by US can integrate muscle strength measures for the detection of subjects at higher injury risk. For instance, associations between muscle size, hamstrings-to-quadriceps ratio, and the hamstring-to-quadriceps strength ratio have been reported previously [28].

### Screening the Athlete for Structural Abnormalities Related to Current or Future Musculoskeletal Complaints

Traditional US and US-based SWE have been adopted as diagnostic tools for the evaluation of tendinopathies, tendon injuries, and ruptures [52]. Indeed, an increased ACSA is generally observed in injured tendons, likely due to an increase in water content alongside hypervascularization [82]. A reduced stiffness pattern evaluated by SWE has been found in pathological Achilles [113–115], patellar [116, 117] and rotator cuff tendons [118, 119]. Similarly, muscle stiffness, related to muscle activity and joint mobility, can be linked to tendinopathy. For example, an increased stiffness of the upper trapezius muscle [120] and a decreased stiffness of the deltoid muscle [121] are associated with rotator cuff tendinopathy. SWE and US may be also useful for the early detection of subclinical tendinopathies, providing additional time for the implementation of conservative measures [52]. Several studies of athletes participating in different sports have shown that tendon softening (i.e., reduced stiffness), detected with SWE, may explain current (and predict future) tendon pain and tendinopathy [116, 122–124].

Several studies have investigated the relationship between lumbar muscle characteristics and low back pain. It has been observed that multifidus ACSA is negatively associated with and predictive of low back pain for up to 12 months in different populations, including athletes [125]. Similarly, paraspinal muscles ACSA was found to predict low back disability (but not pain intensity) in people with low back pain of 2–12 month duration [126]. Other reports show that lumbar muscle stiffness differ in individuals with low back pain compared with asymptomatic controls [127, 128]. Moreover, swimmers with low back pain exhibited higher stiffness of the psoas major and lower stiffness of pectoralis minor compared to a control group [129].

### Monitoring the Return to Sport After a Musculoskeletal Injury

**Fig. 6:**
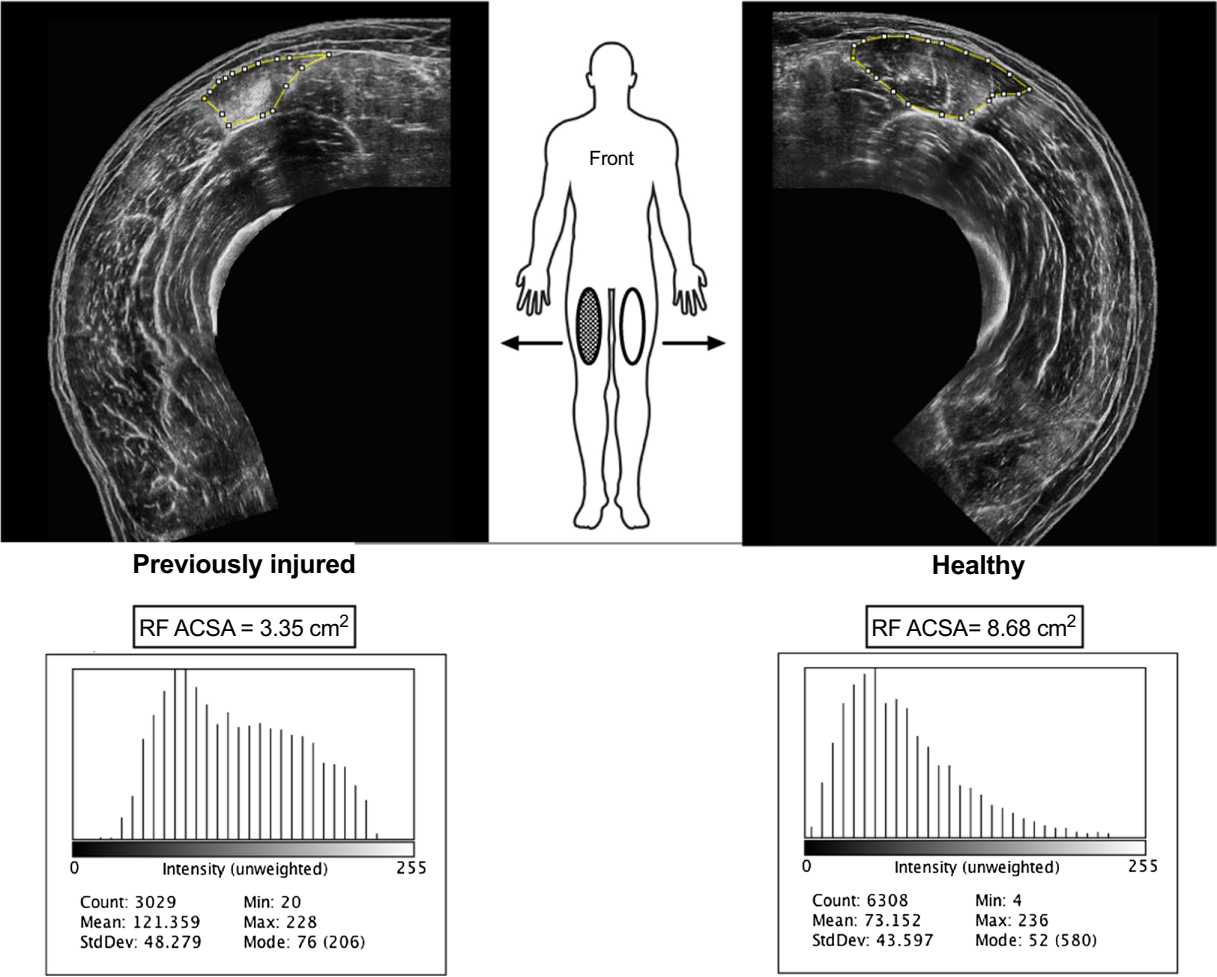
Panoramic scans of right and left quadriceps femoris muscle of a young volunteer who previously sustained a RF muscle injury. Respective RF muscles contours and areas are shown for the previously injured and healthy legs, together with the histogram analyses of echo-intensity (expressed in arbitrary units), suggesting decreased muscle quality in the previously injured leg. Images were obtained using a 4.7 cm linear transducer. *RF* rectus femoris, *ACSA* anatomical cross-sectional area, *StdDev* standard deviation

Loss of muscle structure and function may occur after musculoskeletal injuries and/or post-surgery situations mainly due to the disuse and/or detraining experienced [130, 131]. Therefore, monitoring the change in US-derived parameters allows more specific insight into the actual status of athletes during their return to sport. For instance, muscle ACSA and volume have been assessed in athletes and patients recovering from anterior cruciate ligament reconstruction [132–135] or hamstrings strain injury [136–138]. Such evaluation can be helpful also to examine the effectiveness of rehabilitation programs on muscle morphological outcomes, when comparing them to pre-injury measures [139]. In addition, since muscle architecture is known to be affected by musculoskeletal injuries [105, 140, 141], US can be used to characterize the behaviour of fasicles and PA during the return to sport using pre-injury measures or the contralateral uninjured limb as reference. For instance, after Achilles tendon ruptures, and for the following 2–4 weeks, the injured limb presents altered medial gastrocnemius architecture (shorter Lf and greater PA) compared to the gastrocnemius in the uninjured limb [140]. A representative EFOV transverse scan of the quadriceps muscle group of a subject who suffered a previous muscle injury is presented in Fig. 6.

## Methodological Considerations

### Muscle Ultrasound

#### Muscle Architecture Assessment

The reflection of the acoustic waves is dependent on the orientation of the transducer (i.e., the direction of travel of the sound waves) relative to the position of the underlying tissue [2]. The optimum ‘angle of incidence’ (i.e., the angle at which the sound waves meet the surface of the scanned tissue) is located when the direction of the structure is nearly perpendicular to the direction of motion of the acoustic waves [2]. Thus, during the acquisition of longitudinal scans for the evaluation of muscle architecture, it is crucial to carefully align the transducer with the muscle fascicle plane [1]. For the methodological considerations related to manual digitalization variability and the size of the FOV employed in the assessment of muscle architecture properties, we refer the reader to the sections ‘5.1.4’ and ‘5.1.2’, respectively.

#### Muscle Thickness Assessment

The principal methodological consideration in the assessment of MT is related to the pressure exerted by the operator on the skin and the underlying tissue, which in turn would influence the measurement. As muscle tissue can be deformed, excessive transducer pressure applied by the operator will result in lower MT values. Transmission gel should be used to improve the acoustic contact and to reduce the transducer pressure on the skin.

As previously explained, MT can be evaluated by placing the transducer both transversally [18] or longitudinally [6, 17] with respect to the scanned limb. In our experience, utilising the longitudinal scan to evaluate MT is the better of the two. In transversal scans, muscle borders are curved and thus the evaluation of MT would be entirely dependent on where MT is measured (i.e., between which specific points), thus being more difficult to standardize. Conversely, if the image is correctly acquired in longitudinal scans, the aponeuroses should be parallel (at least in scans performed in the middle of muscle belly, the ROI where MT is usually assessed) and thus the MT will be represented by a simple linear distance between two linear borders. We, therefore, suggest obtaining MT and muscle architecture from the same image to achieve a measure of muscle dimension in relation to the fascicle arrangement analysed. MT values are generally obtained by averaging three measurements across the proximal, central, and distal portions of the acquired image [17].

### Tendon Ultrasound

#### Assessment of Tendon Mechanical Properties

The assessment of tendon mechanical properties is one of the most challenging to implement in a sports scenario, due to several methodological and technical aspects to consider. For an extensive and critical evaluation of the US-based testing of tendon mechanical properties, we refer the reader to Seynnes et al. [45]. However, we will briefly describe some of the possible sources of error below.

The US-based evaluation of tendon mechanical properties are also restricted by narrow FOV. As such it can be challenging to scan both proximal and distal extremities of the tendon simultaneously, an issue which can significantly improve data accuracy. Nonetheless, the use of a longer transducer and the adjustment of transducer orientation to account for the three-dimensional behaviour of tendon deformation can overcome this issue [45]. The acquisition of both extremities enables the use of automated tracking or at the very least semi-automated, which should always be employed to reduce manual intervention variability and operator bias. The use of both tendon insertion points as opposed to related structures such as aponeuroses is strongly recommended [45]. In addition to decreased variability, automated tracking may reduce processing time. To acquire an accurate estimation of tendon force, both internal and external moment arms should be measured individually. Moreover, subjects should be trained to reduce co-contractions and to increase or decrease moment production linearly at a target rate [45]. In addition, subjects should complete preconditioning of the tendon prior to assessment via a minimum of five submaximal contractions [142]. Finally, data acquisition should have high temporal and spatial resolution and should be centralized into a single software. Absence of image lag when acquiring other signals synchronously should be achieved [45].

## General Issues

### Echo intensity Assessment

Echo intensity values rely on grayscale analysis (Fig. 6). For this reason, US settings (e.g., brightness, contrast, number and position of foci, persistence and special views) must be kept consistent between pre- and post-measurements. For the same reason, post-processing scan modifications (e.g., brightness, contrast, colour balance/threshold) should not be applied during analysis.

While some reports support the use of echo intensity as a marker for muscle quality in different interventions [143, 144], in our experience echo intensity measures do not show great sensitivity for changes in muscle quality after training or unloading periods in young subjects (unpublished data). In support of this notion, echo intensity response to chronic exercise training is controversial in young subjects, while decreases in echo intensity seem to be consistent in older adults [33]. Moreover, echo intensity has been shown to change at the presentation of a serious injury (Achilles tendon rupture), but was not different when compared to the contralateral muscle after 2 and 4 week post-injury [140]. Thus, we recommend a careful approach when using this proxy for muscle quality in athletes.

#### Shear Wave Elastography

SWE systems are built on the assumption that the analysed soft tissues are completely elastic, incompressible and isotropic (i.e., obtained values are independent from the direction of measurement). However, muscles and tendon exhibit an anisotropic behaviour. Indeed, it has been observed that shear waves travel at a higher velocity along the direction of the fibres compared to transversally to them [145, 146]. It is, therefore, essential to standardize the transducer's position, so that it is aligned with the orientation of muscle or tendon fibres [48, 49].

Other factors such as transducer pressure, ROI sizes and scan acquisition time can also influence SWE stiffness outcomes [147]. SWE acquisitions on muscle and tendon should be performed with the lightest transducer pressure, a shorter acquisition time, maintaining constant ROI size [147]. In addition, the propagation of the shear waves is attenuated at greater acquisition and is thus dependent on surrounding tissues (e.g., thick superficial fat layers) [48].

#### B-Mode vs. EFOV

The main limitation of standard B-mode US is the relatively small FOV, which is determined by the size of the transducer [1]. The length of the commercially available transducers is typically 4–5 cm, although it may be as large as 10 cm in some devices. Shorter transducers with higher temporal resolution (i.e., acquisition frame rate) are ideal for the study of muscles with short Lf (e.g., triceps surae muscle group). However, a limited FOV can affect the results for Lf values of muscles that have longer fascicles and more complex fascicle arrangement (e.g., quadriceps femoris muscle group) [19, 106]. In these circumstances, longer transducers are advised for the evaluation of muscle architecture.

  If only conventional B-mode is available, Lf can be estimated using extrapolation methods [148, 149]. Aside from the manual linear extrapolation method, one possible approach is represented by a trigonometric equation [148], in which the Lf estimation is based on MT, PA and the angle between the aponeuroses. While this technique makes the analysis less time consuming and thus suitable for an elite sport context, it neglects fascicle and aponeuroses curvature. Therefore, this techniques seems inaccurate for muscles with a non-homogeneous architecture along their length and concave/convex fascicle (e.g., BFlh) [106].

The EFOV technique allows for the study of larger anatomical structures, thus representing a good strategy to overcome the limitation of a restricted FOV of the transducer for the assessment of muscle architecture. This technique is based on texture mapping algorithms that stitch together sequences of images collected during scanning to reconstruct a unique combined panoramic image [21]. EFOV can be used to evaluate both ACSA (performing a transverse scan) and muscle architecture (performing a longitudinal scan) [1]. Longitudinal EFOV scans, compared to B-mode US, have the clear advantage of providing larger images, where no extrapolation is required to assess Lf. This is of particular importance for muscles with long fascicles and where the architectural arrangement changes between different portions of muscle length [148]. On the other hand, one major limitation is the difficulty of keeping the transducer aligned to the fascicle plane throughout whole muscle scans [19]. Transversal EFOV scans include potential limitations such as the pressure applied on the skin by the operator (as explained also in sect. 4.1.2) and in the employment of linear transducers for the assessment of curved surfaces [1]. It is worth noting, however, that not all US devices enable the possibility to perform EFOV scans.

Others technique that obtain larger FOV images, such as dual probe technique (i.e., the use of two linear transducers placed in series) [150] and 3D US [151], have been proposed and employed in a laboratory setting. However, in elite sports scenarios these techniques are difficult to implement due to the need of further expensive instruments (e.g., motion capture systems, more transducers) and algorithms for data analysis and processing.

#### Static vs. Dynamic US

Most US measurements have generally been acquired in resting conditions. However, over the past few decades US has been used also to study muscle and tendon behaviour during dynamic movements [2, 10, 11]. In particular, PA and fascicle, tendon and aponeurosis length changes have been evaluated both during muscle contraction and passive joint movement [10]. B-mode US seems to be a reliable method to evaluate muscle architecture during movement [11].

Since every sports activity is characterized by a combination of movements, and musculoskeletal injuries generally occur during dynamic contractions; these measures obtained by dynamic US might be potentially more informative when employed in a sports context. However, several technical considerations that limit the use of dynamic US in an applied sports setting should be considered. First, dynamic conditions require the transducer to be completely fixed to the limb scanned and this procedure necessarily results in compression of the underlying structures [10]. Secondly, the operators should strive to maintain a consistent alignment of the image plane with the fascicle plane. Indeed, errors in fascicle length measurements due to transducer malalignment issues can reach 20% [152, 153]. The best estimate of muscle architecture seems to be obtained keeping the transducers perpendicular to the skin, at least for gastrocnemius medialis muscle [152, 153]. Whether this alignment is always maintained during a dynamic movement is difficult to determine [2]. Such alignment must be chosen with care as it is known that muscle fascicles arrangement changes with contraction, and so does the shape of the muscle itself. Thus, the best alignment between the transducer and the fascicle plane can be seen as a trade-off between transducer fixation and changes of muscle geometry and shape due to contraction. Furthermore, the visibility of muscle and tendon in their entire length is essential in dynamic conditions, but the limitations related to a restricted FOV are present as discussed for the static condition (see sect. 5.1.2). This is particularly important as muscle and tendon length changes in a small region of interest do not necessarily mirror the behaviour in other regions of the muscle [10]. This limits the number and/or size of the muscles and tendons that can be imaged with this technique [2]. The frame rate (i.e., how many images the US device can acquire in one second) is another important parameter to consider when acquiring dynamic US scans. Indeed, for fast dynamic tasks (i.e., high speed running, jumping, and landing) a high temporal resolution is necessary. Finally, despite the application of automatized feature-tracking algorithms, data analysis may be more complex for dynamic compared to static conditions.

All these factors can be reasonably controlled in laboratory settings if all the recommendations to improve the reliability and validity of dynamic US are followed [11]. However, in a sports context, where time and equipment are often limited, its application may be very challenging. Thus, we believe that teams and sports federations willing to implement US with athletes should focus mainly on static measures until technological advances in dynamic US have been achieved.

#### Operators and Raters Experience

Acquisition of US scans is known to be an operator-dependent procedure. In particular, EFOV scans are more difficult to acquire compared to B-mode US scans, primarily due to the challenge of keeping the transducer parallel to the fascicle plane over a large area of interest [154]. Extensive operator training is, therefore, essential to ensure reliability and to manage all the discussed technical issues (e.g., probe alignment and transducer pressure). For novice operators, we suggest performing a calculation of the repeatability of the measurements (via interclass correlation coefficient and coefficient of variation [CV] of the standard error mean) and the minimal detectable change (MDC, i.e., the minimum difference suggesting a real change, which would not be subjected to repeated measurement errors) before collecting data. Because CVs and MDCs could differ between different muscles, care should be taken in the calculation of such parameters for each US technique and US-based measurement. Sports teams and federations interested in implementing US imaging in an elite sports context should consider the introduction of a specialized individual (a sonographer or a sports scientist /sports medicine specialist with expertise in musculoskeletal imaging) to the staff.

In addition, manual data analysis can be influenced by operator’s experience, specifically the variability related to manual digitalization process. For instance, in a recent study, Franchi et al. found worse agreement between US and MRI values for a completely novice rater compared to a trained rater in the assessment of individual hamstring muscle ACSAs [26]. Interestingly, this agreement was found to improve for the trained rater after manual analysis of MRI scans, highlighting the importance of operator’s training also for data analysis [26]. Due to the impact of operator’s experience and the time requirement to gain this, attempts have been made to automate this data analysis process. In particular, an ImageJ macro tool to automate measurements in B-mode US scans called ‘Simple Muscle Architecture Analysis’ (SMA) has recently been proposed by Seynnes & Cronin [155]. From this preliminary study, this automated analysis seems to not induce any systematic bias when compared to manual analysis [155]. Therefore, this tool seems to be a promising approach to analyse images from superficial muscles in an objective and less time consuming way. However, SMA relies on muscle fascicle orientation, and thus may be not yet appropriate for muscles that present complex and variable muscle architecture arrangement (e.g., BLFlh). To date, no automatized analysis for EFOV images (both for longitudinal and transverse scans) is available.

Although different scanners enable the measurement of US parameters on the machine itself, it is more usual to perform the analysis off-line. Based on our experience, we believe that this second approach makes the analysis more accurate as it allows image modifications post -acquisition, thus facilitating various analyses. Moreover, off-line analysis represents the only solution if the fascicle is not fully visible. Ultimately, since most of the analysis in previous works was conducted using the same software (ImageJ), this can lead to an improved standardization of such analyses within the scientific community.

## Future Directions

The morphological and mechanical properties of muscle and tendon are associated with functional and sports performance. Indeed, they play an important role in testing the response to training, detecting athletes at higher risks of injury, screening athletes for structural abnormalities related to current or future musculoskeletal complaints and monitoring their return to sport after a musculoskeletal injury (Table 1). Despite the well-known problems related to the interpretation of associations suggesting the potential effect of confounders; the results of previous observational studies seem to support the plausibility of a causal link. Thus, regular assessment of these parameters may provide important information for sports practitioners and medical staff working alongside athletes. However, while US provides a non-invasive evaluation of these characteristics, many practical and methodological recommendations should be considered for its implementation in an elite sports scenario. Since extensive training and experience are required for US scan acquisition and analysis, we believe that a new specialized figure in musculoskeletal imaging (a sonographer or a sports scientist with expertise in US) should be introduced (or trained) in teams/federations interested in implementing this technique. Future research should focus on designing new tools for data processing and analysis, with the aim to give real time feedback to the staff and make it less time consuming and more objective (i.e., less dependent on operator’s experience). Automatized programs for the assessment of muscle ACSA and EFOV scans should be developed. Furthermore, appropriate designs and analysis are warranted to understand the prognostic ability of these measures.

**Table 1:** Principal US-based measures of muscle and tendon morphological and mechanical properties, their functional significance and application in sports scenarios, and associated methodological considerations

Parameter	Functional significance	Application in sports scenarios	Methodological considerations
Muscle Architecture (Lf; PA)	Lf is related to velocity of contraction PA is related to maximal force production	Monitoring of athletes’ status Detection of athletes at potential higher injury risk (short Lf)	Transducer alignment Manual digitalization variability Field of view employed Operator’s experience (in particular for the acquisition of EFOV scans)
Muscle dimensions (MT; ACSA; volume)	Related to joint torque production and isokinetic strength	Monitoring of athletes’ status Detection of athletes at higher injury risk (reduced muscle size)	Transducer pressure Longitudinal vs. transverse scan for MT assessment Operator’s experience (in particular for the acquisition of EFOV scans)
Muscle quality (echo intensity)	Proxy for muscle quality/composition	Evaluation of potential alteration post-injury and during the return to sports journey	Maintenance of the same US setting Avoidance of post-processing analysis application Limited sensitivity in changes
Tendon dimensions (thickness; ACSA)	Related to the distribution of stress within the tendon	Detection and evaluation of tendinopathy	Operator’s experience
Tendon mechanical properties (stiffness; strain; stress)	Related to the capacity to transmit muscle force to the bone	Monitoring of athletes’ status Detection of tendinopathy	Scanning issues Tracking issues Estimation of tendon force Signal synchronization
Muscle and tendon stiffness measured with SWE	Related to force production and to the capacity to transmit muscle force to the bone	Monitoring of athletes’ status Indirect assessment of muscle force and joint mobility Detection and evaluation of musculoskeletal complaints	Anisotropic physical properties of skeletal muscle Management of technical factors (transducer pressure; region of interest size; scan acquisition time; acquisition depth)

*MT* muscle thickness, *Lf* fascicle length, *PA* pennation angle, *SWE* shear wave elastography, *EFOV* extended-field-of-view

## Conclusion

**Fig. 7:**
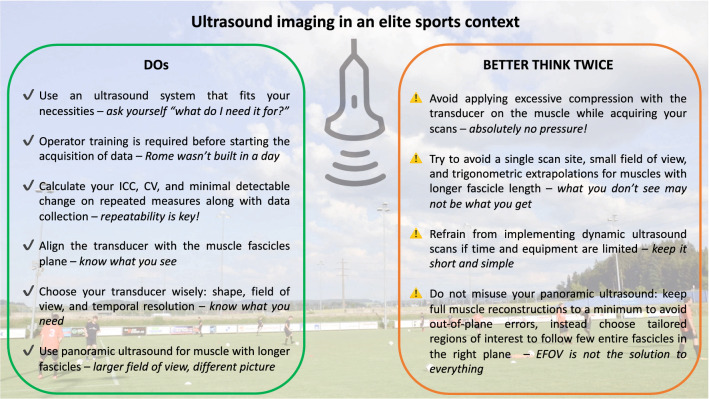
Panel of practical recommendations for the implementation of ultrasound-based imaging in the elite sports context. *ICC* intraclass correlation coefficient, *CV* coefficient of variation, *EFOV* extended-field-of-view ultrasound technique

US may have many promising application areas in an elite sports scenario. However, the practical issues and methodological considerations elaborated in this review should be considered when striving to provide valid and relevant information for benefiting athletes' performance enhancement or for health protection. Moreover, this review may have re-scoped the agenda of potential future research directions in connection with implementing US imaging for the assessment of muscle and tendon properties in elite sports (Fig. 7).
